# Aldehyde dehydrogenase as a marker and functional mediator of metastasis in solid tumors

**DOI:** 10.1007/s10585-015-9755-9

**Published:** 2015-10-07

**Authors:** Mauricio Rodriguez-Torres, Alison L. Allan

**Affiliations:** London Regional Cancer Program, London Health Sciences Centre, London, ON Canada; Department of Anatomy & Cell Biology, Schulich School of Medicine and Dentistry, Western University, London, ON Canada; Department of Oncology, Schulich School of Medicine and Dentistry, Western University, London, ON Canada; Lawson Health Research Institute, London, ON Canada; London Regional Cancer Program, Room A4-132, 790 Commissioners Road East, London, ON N6A 4L6 Canada

**Keywords:** Aldehyde dehydrogenase, Metastasis, Solid tumors, Cancer stem cell, Biomarker

## Abstract

There is accumulating evidence indicating that aldehyde dehydrogenase (ALDH) activity selects for cancer cells with increased aggressiveness, capacity for sustained proliferation, and plasticity in primary tumors. However, emerging data also suggests an important mechanistic role for the ALDH family of isoenzymes in the metastatic activity of tumor cells. Recent studies indicate that ALDH correlates with either increased or decreased metastatic capacity in a cellular context-dependent manner. Importantly, it appears that different ALDH isoforms support increased metastatic capacity in different tumor types. This review assesses the potential of ALDH as biological marker and mechanistic mediator of metastasis in solid tumors. In many malignancies, most notably in breast cancer, ALDH activity and expression appears to be a promising marker and potential therapeutic target for treating metastasis in the clinical setting.

## Introduction

Metastasis is a life-threatening systemic condition, with ninety percent of all cancer deaths resulting from cancer cell dissemination from the primary tumor to distant vital organs [1]. Navigation of the metastatic cascade is a complex, multistep process involving multiple tumor cell phenotypes, body compartments, and accelerated evolutionary cell trajectories [2]. Accordingly, in spite of enormous and earnest progress in elucidating the mechanisms that drive metastasis, the mortality of metastatic cancer has improved very little in the last several decades [3].

Despite the deadly nature of metastasis, it is a remarkably inefficient process. In fact, only a small fraction of cancer cells that survive in the systemic circulation are able to give rise to clinically relevant metastases [4]. Therefore, the identification, isolation, and characterization of potential metastasis-initiating cell (MIC) subpopulations has become a priority for many metastasis research groups including ours. One of the most attractive candidates for MICs are putative cancer stem cells (CSCs), which have been identified in a diverse array of hematopoietic and solid tumor types (reviewed in [5] and [6]). These CSC subpopulations can be defined by their capacity for sustained self-renewal and the ability to give rise to the heterogeneous population of cancer cells that make up a tumor. Importantly, it has also been shown that cells with a CSC phenotype characterized by high aldehyde dehydrogenase (ALDH) activity have an enhanced capacity for metastatic behavior in vitro (adhesion, colony formation, migration, and invasion) and/or metastasis in vivo [7–11], supporting the hypothesis that CSCs might act as the MIC subpopulation.

In the past several decades, increasing evidence has supported the role of ALDH as a biological marker for stem-like cancer cells and aggressive tumor cell behavior, as well as an indicator of poor clinical outcome with particular prominence in breast cancer experimental models and clinical studies (reviewed in [5, 12–15] ). In addition to its role as a detoxifying enzyme and key mediator of stem/progenitor cell expansion and differentiation, the functional and mechanistic involvement of ALDH in tumor initiation and progression has become a topic of considerable interest in the cancer field. While the involvement of ALDH in primary tumor formation, therapy resistance, and malignant behavior in vitro has been extensively described in the literature (reviewed in [5, 12–14, 16] ), the role of ALDH in metastasis has been less evident. The purpose of this review is to highlight the most recent evidence supporting a specific role for ALDH in metastasis, both in pre-clinical mechanistic studies and in vivo models, as well as in the clinical setting. Clarification of the tumor types affected, the isoforms implicated, and the underlying molecular mechanisms of ALDH in driving metastasis is necessary in order to achieve effective translational targeting of this important enzyme.

## The human ALDH superfamily

Nineteen different ALDH functional genes and multiple splice variants have been characterized to date. Although they are widely expressed in multiple different tissues, these ALDH isoforms display tissue- and organ-specific expression patterns and have also been found in various cellular sub-compartments including the cytosol, nucleus, mitochondria, and endoplasmic reticulum (reviewed in [5] ). In these locations, ALDH catalyzes the oxidation of aldehydes to their corresponding carboxylic acids. For example, different ALDH families are capable of detoxifying highly reactive aldehydes that are products of lipid peroxidation (ALDH1, ALDH3, ALDH8) [17–19]. Others are critical regulators of the retinoic acid pathway through involvement in the catalysis of retinaldehyde to retinoic acid, and therefore play an important role in stem and progenitor cell expansion and differentiation (ALDH1A1, ALDH1A2, ALDH1A3) [20]. ALDH also has been found capable of inactivating xenobiotics, including the alkylating agent cyclophosphamide (CP) and analogous chemotherapeutic drugs (ALDH1A1, ALDH3A1) [21]. In addition, it has been observed that ALDH is mechanistically involved in other diverse cell activities including structural and osmoregulatory functions (ALDH1A1, ALDH3A1, ALDH7A1) [14, 22]. Importantly, the ability of ALDH to regulate cell proliferation and self-protection is believed to contribute its involvement in mediating CSC capabilities such tumor progression, phenotypical heterogeneity, and therapy resistance [5].

## Functional role of ALDH in cancer

### ALDH and retinoic acid signaling in cancer cells

The retinoic acid signaling pathway has been implicated in normal and cancer cell function including the regulation of gene expression and development [23–26]. In tumor biology, the retinoic acid pathway appears to play a dualistic role involving epigenetic context-dependent differential gene expression, mediation of apoptotic pathways, and immune system regulation [10, 20]. The human cytosolic ALDH1A subfamily (made up of ALDH1A1, ALDH1A2, and ALDH1A3) irreversibly oxidizes retinaldehyde to retinoic acid (RA). Subsequently, RA is translocated to the nucleus where it is able to regulate the transcriptional activity of more than 500 genes through activation of retinoic acid receptor (RAR), retinoic X receptor (RXR), and nuclear hormone receptors peroxisome proliferator activated receptor beta/delta (PPAR/β/δ). RA exerts differential effects on tumor growth in a cell context-dependent manner. It has been found that retinoic acid activation of RARs and RXRs is followed by cell cycle arrest and differentiation due to increased transcription of tumor suppressor factors, whereas RA activation of PPARs has been found to mediate the increased expression of oncogenes and subsequent cell cycle progression observed in other experimental model systems (reviewed in [20]). In addition, it has been observed that the influence of RA on tumor growth might be mediated by epigenetic silencing of RA-inducible tumor suppressors [10, 27].

Importantly, RA-producing ALDH isoforms, including ALDH1A1 and ALDH1A3 (but not ALDH1A2), are among the most common ALDH isoforms associated with a diverse arrange of hematopoietic and solid tumors. There is accumulating evidence that their increased expression selects for tumor cell subpopulations exhibiting stem-like or aggressive tumor cell phenotypes, indicating that the tumorigenic “branch” of the retinoic acid pathway might be co-opted in some tumors and/or that other non RA-dependent functional roles of ALDH1A1 and ALDH1A3 might be involved in tumorigenesis [7, 9, 10, 28]. For example, ALDH1A1 and ALDH1A3 have been shown to play functional roles in lung and breast cancer cell migration and invasion, although the mechanisms underlying this behavior have not been established [10, 29, 30]. It is of note that RA has been used to dramatically improve clinical outcome in patients with acute promyelocytic leukemia (APL); where ninety-eight percent of patients with this disease carry a fusion of the PML and RARα genes that impairs the ability of RARα to induce hematopoietic stem/progenitor cell differentiation at physiological levels of RA [31, 32]. However, attempts to use RA to induce tumor cell differentiation in other cancer types have shown mixed results at best in clinical trials (reviewed in [20] and [16]). The potential causes of the disparate results obtained after targeting the retinoic acid pathway in cancer are discussed later in this review.

### ALDH plays a self-protective role in cancer cells

There is increasing evidence implicating ALDH in cancer cell self-protection against both endogenous and exogenous threats. Antioxidant and substrate-specific drug inactivation are among the key mechanisms underlying these capabilities. For example, ALDH has a NADPH recycling function that is believed to support antioxidant cell capabilities. In addition, it has been observed that ALDH is often co-expressed with antioxidant factors and drug efflux channels in cells with high ALDH activity [22, 33–39]. ALDH1A1 and ALDH1A3 are capable of enzymatic inactivation of alkylating agents such as CP and other oxazaphosphorines [40–43]. Moreover, ALDH appears to confer resistance to drugs other than CP and analogues including doxorubicin, cisplatin, arbinofuranosyl citidine (Ara-C), temozolemide and taxanes [28, 44–47], although the mechanisms underlying this are less clear. A comprehensive review of ALDH involvement in chemotherapy and radiotherapy resistance has recently been published by Januchowski et al. [21]. Thus, ALDH is not only involved in physiologic and cancer cell proliferation and differentiation, but also in promoting tumor cell survival through direct inactivation, indirect expulsion of xenobiotics, and enhancement of the oxidative stress resistance response.

## ALDH as a marker for cancer stem cells

### Assessment of ALDH in cancer research

Different approaches have been used to quantify ALDH in cancer cells and tumor tissue. Early methods for determining ALDH levels relied on measuring enzyme kinetics or immunoblotting of enzymes released after cell lysis, in addition to immunohistochemical detection. However, these methods are flawed by antibody cross-reactivity between enzyme isoforms and enzyme structural instability [48, 49]. An alternative and more recent approach consists of measuring ALDH activity in viable cells by quantifying the ALDH-mediated intracellular retention of the fluorescent compound BODIPY-aminoacetate (BAA^−^) using flow cytometry-based methods [7, 23, 50–52]. This functional assay is commercialized as the ALDEFLUOR™ assay and depends on the conversion of the uncharged ALDH substrate BODIPY amino acetaldehyde (BAAA), which freely diffuses in and out of the cell, into the negatively charged BAA-compound. As a result, after addition of BAAA, cancer cell subpopulations with elevated activity of ALDH (ALDH^hi^) become highly fluorescent and can be identified using flow cytometry gating criteria. The ALDH^hi^ cell subset can be distinguished when compared to cells treated with the ALDH1 inhibitor 4-(diethylamino) benzaldehyde (DEAB), which is used as a reference control. Although the ALDEFLUOR™ assay works well in various human models, there is controversy about its adequacy for identifying cancer stem cells in murine models given disparate results among groups isolating murine HSCs using this technique [53–56]. However, enrichment of murine CSCs have been recently reported using the ALDEFLUOR™ assay in two different models of mouse breast cancer [57, 58]. In addition, attempts at in vivo stem cell labeling have employed ALDH radiolabeled substrates. However, the resultant charged compounds were not polar enough to be retained into the cells [59].

### ALDH isoforms involved in the ALDEFLUOR™ assay

Early reports identified ALDH1 as the main ALDH enzyme family responsible for the enzymatic activity reported using the ALDEFLUOR™ assay [52, 60]. However, recent controversy has emerged regarding the family and subfamily isoforms acting on the BAAA substrate and thus responsible for the readout in this assay. This distinction is of importance given the ample and tissue-specific distribution of ALDH isoforms in normal tissue, the reliance on the ALDEFLUOR™ assay for isolation of viable CSCs for subsequent downstream assessment, and differential roles of different ALDH1 isoforms in tumorigenesis and metastasis [10]. In breast cancer, mixed results have been reported regarding the specific ALDH1A isoenzyme involved in tumorigenesis and the ALDEFLUOR™ assay, with some groups reporting ALDH1A1 as the main enzyme involved and others showing that ALDH1A3 is the accountable isoform [9, 10, 61, 62]. Recent research by our group using human breast cancer cell lines indicate that although both isoforms are involved in different phases of the metastatic cascade, 50 % of the ALDEFLUOR™ activity is driven by the ALDH1A3 isoform with no significant participation of ALDH1A1 in this assay (*our unpublished data*). In addition, other studies have found enzymatic participation of ALDH1A1, 1A2, 1A3, 2, 7A1 and 8 in the ALDEFLUOR™ assay in other tumor types [22, 63–65]. Thus, multiple ALDH isoforms may contribute to both the readout of the ALDEFLUOR™ assay and to cellular function in tumorigenesis in a tissue-specific manner.

It is important to clarify that, by current nomenclature, the term ALDH1 does not accurately describe any of the isoforms of the ALDH superfamily [12] and could refer to ALDH1A1, ALDH1A2, ALDH1A3, ALDH1B, ALDH1L1, or ALDH1L2. This is a major problem in the ALDH/cancer stem cell literature, where ALDH1A1 is often used interchangeably with ALDH1 or ALDH. Further confusion arises when ALDEFLUOR™ positivity is referred to as ALDH1 activity or positivity. To address this ambiguity, in the current review we will refer to ALDEFLUOR™ positive cells or ALDEFLUOR™ activity whenever the source refers to a phenotype identified using the ALDEFLOUOR™ assay. Notably, Pors et al. [12] recently reported that the specific isoform identified using BD Biosciences clone 44 or Abcam ab52492 antibodies against ALDH1 is ALDH1A1. In this review, we will therefore clarify this whenever ALDH1 expression is reported by authors using either of these antibodies. In addition, if the isoform specificity of the ALDH1 antibody could not be established, this will also be noted.

### Recent evidence supporting ALDH as a CSC marker

Multiple markers have been used for enriching cells with stem-like properties from human tumor primary tissue and human cancer cell lines, and this is reviewed exhaustively elsewhere in the literature [6, 66–70]. We have previously reviewed the relevance of ALDH as a marker for normal and cancer stem cells [5]. Thus, for this review we have summarized the latest findings regarding the importance of ALDH as a cancer stem cell marker since 2010 (summarized in Table 1).

**Table 1:** Recent evidence supporting ALDH as a marker of CSCs and cancer progression

Tumor Type	ALDH isoform	Method of ALDH assessment	Functional/mechanistic observations associated with ALDH	Clinical/prognostic observations associated with ALDH
Breast cancer	ALDH [9, 78, 87, 136, 148, 149] ALDH1 (ALDH1A1 as per [12] ) [78, 87, 119, 135, 149, 180, 181] ALDH1A1 [61, 62, 147] ALDH1A3 [9, 10, 182, 183]	ALDEFLUOR™ [9, 78, 87, 136, 148] IHC [9, 10, 61, 62, 78, 87, 119, 135, 149, 180–184] Immunoblotting [147] qPCR [9, 10]	Increased Notch and β-catenin levels, increased Ki67 [148] Increased HIF-1/2α expression [149] Increased HOXA1 and MUC4 expression [10] Increased in vitro invasion [10, 78], growth in 3D Matrigel™ [78], Increased primary tumor engraftment in patient derived xenograft (PDX) models [87] ALDH1A1 deacetylation regulated by Notch increases tumorigenicity in vivo [147] Dual role of ALDH1A3 in tumorigenicity and metastasis in vivo depending on epigenetic landscape [10]	Poor clinical outcome alone [10, 135, 136, 149] or combined with CD44^+^CD24^−^ phenotype [180] Increased ALDH expression in metastases vs. primary tumors [119] Tumor recurrence [181] and/or metastasis [9, 183] More common in younger woman [184] and those with TNBC [10, 62, 182, 184] Increased tumor stage [9, 62, 182] and/or grade [9] Poor response to chemotherapy [62] Familial risk for breast cancer [61]
Ovarian cancer	ALDH [79, 143, 185] ALDH1 [89] ALDH1 [80] (no isoform annotated) ALDH1A1 [90, 143]	ALDEFLUOR™ [79, 80, 89, 90, 143] IHC [80, 185] Immunoblotting [143] qPCR [90, 143]	Increased in vitro growth, migration, invasion [79, 80], colony formation, resistance to hypoxia [19] and therapy [79, 143], sphere-formation [80, 90], dormancy [143], DNA repair signalling [143], expression of β-catenin [90] Increased tumorigenicity in vivo [79, 80, 89, 90] Increased sphere formation, regulation of FoxM1 and Notch 1 [89]	Poor clinical outcome [80, 143, 185]
Brain cancer	ALDH [91] ALDH1 [186] ALDH1 [92] (ALDH1A1 as per [12] ) ALDH1A3 [108, 187]	ALDEFLUOR™ [91, 186] IHC (93) qPCR [108] DNA methylation profiling [187]	Increased in vitro sphere formation [91, 186] asymmetric division [91, 92], growth [108], therapy resistance [108] Increased tumorigenicity in vivo [91, 92, 108]	Poor clinical outcome [187]
Bone cancer	ALDH [85]	ALDEFLUOR™ [85]	Increased therapy resistance [85]	Increased metastases [85]
Prostate cancer	ALDH [11, 83, 86, 188] ALDH7A1 [11] ALDH3A1 [109]	ALDEFLUOR™ [11, 83, 86, 188] IHC [11, 109]	Increased in vitro colony-forming ability [11, 188], invasion [11, 83], sphere formation [11, 109], migration [11, 83] and therapy resistance [86] TGF-β [11] and Wnt/β-catenin [86] induce ALDH activity Increased in vivo tumorigenicity [11, 86, 188]	Increased expression in metastases [109]
HNSCC	ALDH [93, 189] ALDH1 [189] (ALDH1A1 as per [12] )	ALDEFLUOR™ [93, 189] IHC [189]	Increased in vitro sphere formation [93] Increased Nanog, Oct-3/4, and Stella expression [189] Increased Bmi-1 and Snail expression [93] Increased tumorigenicity in vivo [93, 189]	Poor clinical outcome [93]
Colorectal cancer	ALDH [95] ALDH1 [94, 122] (ALDH1A1 as per [12]) ALDH1 [120, 190] (No isoform annotated)	ALDEFLUOR™ [95] IHC [94, 120, 122, 190]	Increased in vitro sphere formation [94, 95], in vitro colony formation [94] ALDH expression is correlated with β-catenin expression [122]	Poor clinical outcome [94, 122, 190] Loss of ALDH expression in advanced stage disease [120]
Lung cancer	ALDH [96–98, 100] ALDH1A1 [29, 100, 101, 105, 191] ALDH7A1 [192]	ALDEFLUOR™ [96–98, 100] IHC [29, 100, 101, 105] Immunoblotting [191, 192]	Increased in vitro colony formation [29, 100], sphere formation [96–98] and migration [29] Increased in vivo tumorigenicity [29, 97, 100] Increased therapy resistance [105] Increased Notch [100, 101], Rac1 signaling [98] and RhoA/Rock signaling [191] Increased SOX2, CXCR4 and SDF-1 expression [96] Salinomycin targets ALDH^hi^ cells and reduce xenograft growth [96]	ALDH1A1 correlated with poor clinical prognosis [29, 100 Increased recurrence [192]
Cervical cancer	ALDH [99]	ALDEFLUOR™ [99]	Increased colony formation and sphere formation [99] Increased Twist 1, Twist 2, Snail 1, Snail 2 and Vimentin expression [99]	Not assessed
Melanoma	ALDH [65, 106, 193] ALDH1A1 [65, 106] ALDH1A3 [65]	ALDEFLUOR™ [65, 106, 193] Immunoblotting [106] qPCR [65]	Increased in vivo tumorigenicity [65, 106, 193] Increased therapy resistance [65, 106] Increased RA response and stem cell gene expression [65]	Not assessed
Endometrial cancer	ALDH1 [150] (ALDH1A1 as per [12] )	ALDEFLUOR™ [150] IHC [150]	Nodal inhibited ALDH expression trough ubiquitin–proteasome pathway [150]	Not assessed
Renal cancer	ALDH1 [142] (no isoform annotated) ALDH1A1 [104]	IHC [104, 142] Immunoblotting [104] qPCR [104]	Increased invasion [104] Increased therapy resistance [104] Increased in vitro tumorigenesis [104]	Increased tumor grade [142] Poor clinical outcome [104]
Pancreatic cancer	ALDH1A1 [103] ALDH1A3 [107]	qPCR [103] Chromatin immunoprecipitation [107]	Increased colony formation [103] TGF-β/Smad4 inhibition of ALDH transcription [103] Correlation with cell adhesion, growth factor and receptor activity, transcription and differentiation [107]	Not assessed
Hepatobiliary cancer	ALDH [151] ALDH1A3 [194] ALDH3A1 [110]	ALDEFLUOR™ [151] IHC [110, 194]	Increased in vitro cell proliferation [151] TGF-β induces ALDH expression [151] Increased in vivo tumorigenicity [151] Increased Wnt/β-catenin activity [110]	Poor clinical outcome [151, 194] No correlation with clinical outcome [110]
Oesophageal cancer	ALDH [82, 102] ALDH1 [82] (no isoform annotated) ALDH1A1 [102]	ALDEFLUOR™ [82, 102] IHC [82, 102]	Increased sphere formation, therapy resistance and SOX9 and YAP1 gene expression [82] Increased colony formation and invasion [102] Increased expression of VIM, MMP2, MMP7 and MMP9 [102] Decreased expression of ECAD [102] Increased tumorigenicity in vivo [102] Increased therapy resistance [82]	Poor clinical outcome [102]

## ALDH and metastasis

While there is growing evidence supporting the use of ALDH as a CSC marker and implicating it as having an important functional role in tumor cell self-protection, differentiation, expansion, and therapy resistance, less is known about its functional role in mediating metastasis. In this section we will review the mechanistic involvement of ALDH in metastasis based on experimental evidence derived from assessment of in vitro of cellular behaviors contributing to metastasis, in vivo animal models of metastasis, and patient-derived metastasis samples.

### Functional association of ALDH with in vitro cell behaviors related to metastasis

Different in vitro assays have been used to model and estimate potential metastatic activity in vivo [71]. Clonogenic assays involving loss of substrate adherence or serum-free media, including colony formation in soft agar and tumorsphere growth in ultra-low attachment plates, are correlated with tumorigenicity and stemness. Therefore, they are often used to evaluate the capability of cancer cells to initiate metastatic growth in vivo [72–75]. Assessments of migration and invasion in vitro have also been used to estimate metastatic potential in vivo [71, 72]. In addition, given the intrinsic resistance of metastases to chemotherapy and radiotherapy, in vitro assessment of therapy response has also proven to be useful [72, 74, 76, 77]. ALDH and several of its isoforms have been recently evaluated for and positively correlated with multiple in vitro cell behaviors that are surrogates of metastatic behavior in vivo.

Tumor cells displaying high ALDEFLUOR™ activity have been demonstrated to have enhanced motility and ability to invade through a 3D basement membrane in breast cancer [7, 78], ovarian cancer [79, 80], osteosarcoma [36, 81], esophageal cancer [82], and prostate cancer [11, 83]; as well as increased therapy resistance in breast cancer [28, 84], ovarian cancer [79], osteosarcoma [85], and prostate cancer [86]. ALDEFLUOR™-positive cells have also been reported to exhibit increased capacity to form tumorspheres in breast cancer [8, 15, 52, 87], human malignant fibrous histiocytoma (HMFH) [88], ovarian cancer [80, 89, 90], brain tumors [91, 92], prostate cancer [11], head and neck squamous cell carcinoma (HNSCC) [93], colon cancer [94, 95], non-squamous cell lung cancer (NSCLC) [96–98], esophageal cancer [82], and cervical cancer [99]. Interestingly, ALDH-positive cells display additional stem-like behaviors such as resistance to hypoxia in ovarian cancer [79] and the capacity for asymmetric division in brain tumor cells [91].

Several specific ALDH isoforms have been correlated with in vitro metastatic behavior. For example, ALDH1A1 expression has been reported to correlate with increased in vitro clonogenic activity in NSCLC [29, 100, 101], esophageal cancer [102], ovarian cancer [90], pancreatic cancer [103], and renal cancer [104]. This ALDH isoenzyme has also been correlated with increased migratory capabilities in lung cancer [29], renal cancer [104], and esophageal cancer [29] and in vitro therapy resistance in lung cancer [105], melanoma [65, 106], and renal cancer [104]. Other ALDH isoforms have been mechanistically associated with metastatic behavior in vitro, including ALDH1A3 in breast cancer [9, 10], melanoma [65], pancreatic cancer [107] and brain cancer [108]; ALDH3A1 in prostate cancer [109] and liver cancer [110]; and ALDH7A1 in prostate cancer [11]. In summary, increasing evidence from in vitro studies suggests a mechanistic role for ALDH in metastasis and have laid the groundwork to further the study of the involvement of this enzyme in metastatic activity in vivo.

### Functional association of ALDH with in vivo metastasis

Croker et al. [7] and Charafe-Jauffret et al. [111], using the ALDH^hi^CD44^+^CD24^−^ and ALDH^hi^ and phenotypes respectively, provided the first direct experimental evidence implicating ALDH^hi^ cells in breast cancer metastasis in vivo. Moreover, it was shown that ALDH^hi^CD44^+^CD24^−^ cells were the only cancer cell subpopulation capable of metastasizing beyond the lungs in a pattern that mirrors the clinical behavior of breast cancer [7]. Concurrently, it was also reported that ALDH1A1^+^CD44^+^CD24^+^ cells correlated with metastatic activity in matched primary and metastatic samples from pancreatic cancer [112]. Subsequent studies have shown that high ALDEFLUOR™ activity enriches for cells with increased metastatic capability in vivo in prostate cancer [83, 113, 114], breast cancer [15, 33], HNSCC [93], osteosarcoma [81], esophageal cancer [102], ovarian cancer [79], hepatic cancer [95], and adenoid cystic carcinoma [115]. More recently, other studies have further investigated the specific ALDH isoenzymes correlated with metastatic activity in vivo. For example, ALDH7A1 has been implicated in prostate cancer metastatic activity after left ventricular injection (LVI) of a prostate cancer cell line [11], while ALDH3A1 has been shown to be associated with metastasis using a mouse tail vein injection model [109]. ALDH1A3 has been observed to be involved in metastasis in vivo in breast cancer after cancer cell xenotransplantation into mouse models [10]. It has also been reported that inhibition of ALDH1A1 by RNA interference in melanoma cell results in reduced metastatic ability in vivo [106], providing functional validation and indicating that ALDH is not only a biological marker for enhanced metastatic ability, but also plays a functional role in metastasis in vivo. Interestingly, in one study, ALDH1A3 was found to promote or inhibit breast cancer metastasis depending on the specific epigenetic context framing retinoic acid signaling in vivo [10]. Another elegant study reported that immune targeting of breast CSCs cancer stem cells using ALDH1A1-specific CD8+ T cells resulted in reduced metastatic activity of breast cancer cells in vivo [116]. Interestingly, this study reported that ALDH1A1 was the main ALDH isoenzyme determining of ALDEFLUOR™ assay activity.

### Association of ALDH and metastasis in clinical tissue samples

The association of ALDH and metastases in the clinical setting has been of interest since the late 1980s, when Marselos et al. showed increased enzymatic activity of ALDH (no isoform specified) in metastatic lesions from colon cancer compared with normal adjacent tissue [117]. This study would contrast with another early publication indicating that ALDH activity was not elevated in the serum of patients with metastatic hepatic cancer when compared with serum of patients with non-metastatic cancers [118]. ALDH expression has been assessed in matched primary tumor and metastases tissues in breast cancer [119], colorectal cancer [94, 120, 121], pancreatic cancer [112], and hepatic cancer [95]. The majority of these reports have shown a positive correlation between ALDH1 expression (ALDH1A1 as per [12] ) and metastasis. However, these results should be interpreted with caution due to technical and ethical limitations of working with metastatic tissue, including small numbers of samples analyzed and lack of consistency in staining and scoring methods. For example, diverse grading scales have been used in these studies to score ALDH staining in pathological specimens, including dichotomous scales and continuous scales with diverse cutoff points. This lack of consistency contrasts with the standardized in vivo functional evaluation of ALDH activity using the ALDEFLUOR™ assay. In a recent study using matched colon primary tumor and metastases, Fitzgerald et al. reported ALDH1 (ALDH1A1 as per [12] ) as a predictor of poor clinical outcome [94], in direct contrast with an earlier study that had found a negative correlation [120]. In a study that might shed light in this controversy, using non-matched primary tumor and metastases in colorectal cancer tissues, only homogeneous and intense expression of ALDH1A1 was correlated with metastasis [122].

In addition to ALDH activity/expression in tumor cells, ALDH expression has been recently also been observed in tumor-associated endothelial cells (TEC) from melanoma and oral cancers in vivo. TEC were shown to exhibit increased expression of angiogenic factors and angiogenic behavior in vitro, suggesting that there might be an ALDH-mediated CSC activity in the tumor vascular compartment which in turn could promote tumor cell escape to the intravascular space [123].

### ALDH in circulating tumor cells (CTCs) and disseminated tumor cells (DTCs)

The detection of circulating tumor cells (CTCs) in blood and disseminated tumor cells (DTCs) in bone marrow has been associated with the presence of clinically relevant metastatic disease and poor clinical outcome for a diverse group of solid tumors [124–127]. However, given the high inefficiency of the metastatic cascade, it has been hypothesized that CTCs and/or DTCs may contain sub-populations of cells with enhanced capacity to initiate and maintain growth and give rise to clinically relevant metastases. The biological characteristics and markers of metastasis-initiating CTCs and DTCs are not completely understood. The preclinical and clinical data reviewed above indicate that ALDH is functionally involved in metastatic activity and is a determinant of cancer clinical outcome, and thus implicate ALDH as a potential biomarker to be assessed in correlation with CTC/DTC activity.

To our knowledge, the first attempt to assess ALDH activity in the systemic circulation in correlation with metastasis was published by Jelski et al. [118]. Evaluating serum alcohol dehydrogenase (ADH) and ALDH activity in a small sample of patients with metastatic hepatocarcinoma, this study found a correlation for ADH, but not for ALDH, with metastatic disease. However, early and subsequent attempts at identifying ALDH expression in CTCs/DTCs sub-populations have been successful in breast and endometrial cancer [128–133]. While the scope of these studies is complex given the multi-compartment distribution of CTCs/DTCs, in general they evaluate one or more of the following variables: (i) expression of ALDH in primary tumors in correlation with CTCs/DTCs, (ii) expression of ALDH in CTCs/DTCs as a stem cell marker and its correlation with clinical prognosis and metastatic activity; and/or (iii) co-expression of ALDH with other known markers of stem-like behavior and cancer progression in CTCs/DTCs. Interestingly, the results of these studies are mixed, but this is not surprising given the nascent nature these type of studies, the small cohorts evaluated, the diversity of assays and parameters used to identify and isolate CTCs/DTCs, the inherent technical difficulties of isolating rare cells form clinical samples, and uncertainty about the biological characteristics of clinically relevant CTCs/DTCs that would allow their identification.

In one study involving 502 non-metastatic breast cancer patients, although ALDH was expressed in CTCs and DTCs in 14 % of patients in the study cohort, it was not correlated with clinical prognosis or metastasis. In addition, this study showed no correlation of EMT markers in CTCs/DTCs with clinical outcome [134]. In another study, ALDH expression in primary breast tumors was not found to be correlated with the presence of CTCs, although it was correlated with clinical outcome in patients with non-metastatic disease [135]. In an additional cohort of non-metastatic breast cancer patients, although ALDH expression in DTCs was correlated with use of neoadjuvant chemotherapy and high tumor grade, it was not associated with metastatic recurrence [130]. Another study in a non-metastatic breast cancer cohort showed that the CD44^+^CD24^−^ phenotype in DTCs was a better clinical predictor of cancer progression relative to ALDH expression [136]. In general, studies evaluating ALDH expression in CTCs/DTCs from patients with early non-metastatic breast cancer have shown less correlation of ALDH in CTCs/DTCs with clinical outcome and metastasis than studies performed in patients with metastatic disease [128, 129]. Therefore, although there is evidence supporting ALDH as a marker of CTC and DTC activity in advanced breast cancer, the functional and clinical implications of ALDH expression in CTCs/DTCs and eventual metastasis in breast cancer patients with early disease remains to be established. This suggests that the use of ALDH in CTCs/DTCs as a risk stratification marker might not be useful in patients with non-advanced disease. However, as described below, important technical considerations involving the methods to enrich CTCs/DTCs might change these results.

It is very important to note that in all the studies mentioned above, CTCs/DTCs were enriched using epithelial markers as primary criteria for their isolation. However, there is increasing evidence suggesting that a substantial proportion of CTCs/DTCs might display a more mesenchymal and aggressive phenotype that may not be picked up by standard CTC/DTC assays [124]. In fact, it has been shown that mesenchymal markers are over-expressed in CTCs and DTCs of metastatic and aggressive subtypes of breast cancer [137, 138]. Moreover, ALDH expression in CTCs has been correlated with poor clinical outcome, metastatic progression, and therapy response in patients with metastatic breast cancer [128, 129]. Interestingly, Liu et al. observed that breast CSCs transition between mesenchymal an epithelial states in order to fully develop metastasis, with ALDH participating during the epithelial and more proliferative phase of metastatic colonization in distant tissues. This suggests that ALDH may not be a key marker expressed during the intravascular and more mesenchymal phase of the metastatic cascade [139, 140]. Overall, the study of ALDH as a biomarker for metastasis-initiating cells and clinical outcome in CTCs/DTC is an emerging and promising field of research that is evolving at the pace of the refinement and validation of the technologies used to isolate this important group of cells navigating the metastatic cascade in the systemic circulation.

### Molecular mechanisms associated with ALDH in metastasis promotion

**Fig. 1:**
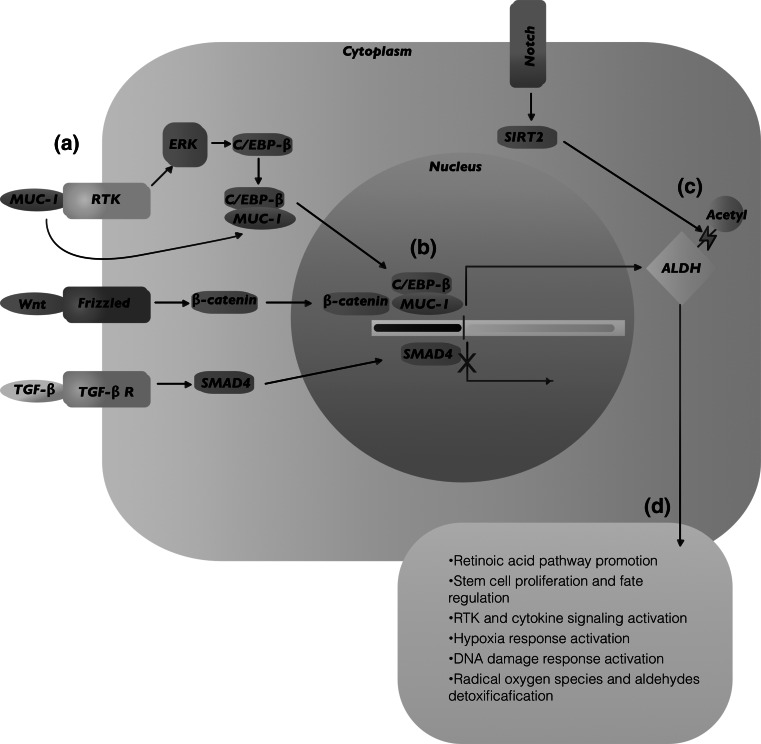
Molecular mechanisms associated with ALDH in metastasis promotion. The molecular mechanisms underlying the increased tumorigenicity and metastatic capacity of ALDH^hi^ cancer cells involve diverse co-expressed molecular factors and signaling pathways. For example, in breast, ovarian, and pancreatic cancer, ALDH1A1 transcription has been shown to be regulated after binding of C/EBPβ, β-catenin, or Smad-4 to the ALDH1A1 promoter sequences (*a, b*). In breast cancer cells, Notch-induced deacetylation of ALDH1A1 can result in increased CSC capabilities (*c*). Taken together, these pathways influence downstream functional behaviors such as stem cell-related decisions regarding proliferation and cell fate, epithelial-to-mesenchymal transition, retinoic acid synthesis, hypoxia, DNA damage response, cytokine and RTK signaling activation, and cell migration (*d*), all of which may contribute to the role of ALDH^hi^ cancer cells in metastasis promotion

The study of the molecular mechanisms underlying the increased tumorigenicity of ALDH^hi^ cells has revealed diverse co-expressed molecular factors and signaling pathways that might potentially also explain the observed increased metastatic behavior of ALDH^hi^ cancer cells. Since 2007, when the Hedgehog pathway was shown upregulated in ALDH^hi^ pancreatic cancer cells [141], there has been accumulating evidence of signaling pathways associated with ALDH^hi^ cells and their malignant activity (Summarized in Fig. 1). In particular, pathways involved in stem cell proliferation and cell fate [93, 101, 110, 141–147]; EMT [11, 36, 78, 81, 86, 90, 100, 110, 112, 146–155]; retinoic acid pathway [10, 89]; hypoxia [33, 79, 149, 156] and DNA damage responses [143, 157, 158]; cytokine and receptor tyrosine kinase (RTK) [158–160] signaling activation; and cell migration [161], among others, have been implicated in promotion of aggressive behavior in ALDH^hi^ cells. Different isoforms of the Notch receptor have been found to be upregulated in ALDH^hi^ cells from breast cancer, ovarian cancer, lung cancer, and osteosarcoma [81, 89, 100, 144, 145, 147, 148]. In addition, ALDH1A1 has been found to be correlated with Notch expression in lung and breast CSCs [100, 147]. The Wnt-β-catenin pathway has been shown to be activated in cancer cells with high expression of ALDH1A1 and ALDH3A1 in prostate, ovarian, and liver cancer cells [86, 90, 110]; and the TGFβ pathway has been reported to facilitate therapy resistance in ALDH^hi^ breast cancer cells, and to be involved in expression of ALDH1A1 in cholangiocarcinoma and pancreatic cancer [90, 103, 151, 152].

Interestingly, for some of these signaling pathways, the evidence suggests dualistic roles in metastasis depending on the cell context. For example, Li et al. reported that ALDH1A1 was involved in lung CSC activity via suppression of the Notch/CDK2/CCNE pathway [101], and, in other study, it was found that Nodal had a tumor suppressor function in ALDH^hi^ cells via the activation of TGFβ pathway [150]. It is of note that there is evidence supporting a more complex model of metastasis navigation in which not only the EMT process but also the mesenchymal-to epithelial-transition (MET) processes are both critical for forming clinically relevant metastases [139, 140]. In this regard, research from two independent groups has shown that ALDH may be a marker and potential mechanistic promoter of a more proliferative/epithelial CSC phenotype via MET, while CD44 may, in contrast, be a marker of a more invasive subpopulation of migratory/mesenchymal CSCs via EMT [78, 146]. In addition, Marcato et al. have reported that ALDH1A3 may have a dual role in breast cancer metastasis promotion depending in the epigenetic cell context through differential retinoic acid signaling [10].

### Therapeutic potential of targeting ALDH in metastasis treatment and prevention

Although there are relatively few studies assessing the impact of pharmacological or immune targeting of ALDH on metastasis in vivo, the majority of them are consistent in reporting a decrease of metastatic burden after targeting of ALDH. It has been shown that after treatment with the ALDH inhibitors DEAB and the novel A37 compound, there is a decreased metastatic activity in murine models of breast and ovarian cancer [33, 90]. It has also been demonstrated that CSC targeting with ALDH1A1-specific CD8^+^ T cells is followed by decreased spontaneous metastatic burden of HNSCC, pancreatic, and breast cancer cells in vivo [116]. Treatment with RA results in downregulation of ALDH1A1/ALDH3A1 expression or decreased ALDEFLOUR™ activity [28, 42, 89], and, in one recent study it has been reported that there is a reduction of in vitro metastatic behavior and reduction of xenograft growth of ovarian cancer cells after RA treatment [89]. However, Marcato et al. have reported that RA treatment has dual effects in a mouse model of human breast cancer metastasis depending on the cell epigenetic context [10].

The small molecule compound disulfiram has been found to display tumor inhibitory activity attributed to different properties, including ALDH1A1 inhibition [35, 162, 163], inhibition of proteasome activity [164, 165], prevention of NF-κB translocation [165, 166], induction of reactive oxygen species generation [167, 168], blockade of the PI3K/PTEN/AKT signaling pathway [169], inactivation of tumor-associated enzymes [170–172], and suppression of metastasis-associated gene expression [170, 171, 173]. Interestingly, it has been reported that disulfiram may have a therapeutic role in the metastatic setting. In one study, cell growth of metastatic osteosarcoma patient-derived cells was significantly decreased after disulfiram treatment in vitro [85]. In another study, cell growth was significantly decreased after disulfiram treatment of a metastatic murine osteosarcoma cell line in vitro [36]. It has also been reported that treatment with this compound decreases spontaneous lung metastatic burden in a syngeneic pre-clinical model of metastatic murine breast cancer [174]. In addition, a recent Phase IIb clinical trial found that addition of disulfiram to chemotherapy was well tolerated and appears to improve survival in newly diagnosed patients with metastatic non-small cell lung cancer [175]. Currently two clinical trials are evaluating the effect of disulfiram in treating glioblastoma multiforme (NCT01907165 and NCT01777919; www.clinicaltrials.gov).

The multiplicity of ALDH isoforms and its widespread tissue/tumor distribution, combined with differential epigenetic landscapes and inconsistency among parameters evaluated in small cohort trials may explain discrepancies observed between preclinical and clinical outcomes. Moreover, selective druggability of different ALDH isoforms is a formidable pharmacological challenge that may also contribute to conflicting results. Physiological concentrations of ALDH are most highly expressed in kidney and liver, which in turn limits the use of nonspecific inhibitors of ALDH that may result in toxic side-effects in patients. In addition, the enzymatic oxidative reaction carried out by different ALDH isoforms is highly nonspecific. For example, the ALDH substrate BAA has been found to be metabolized by different ALDH isoenzymes, including ALDH1A1, 1A2, 1A3, 2, 3A1, and 7 [4, 39, 67, 85]. Thus, there are important biochemical barriers to device a specific ALDH family or isoform inhibitor.

Rationalized small molecule discovery has been proposed as a viable methodology to surmount these difficulties and has been successfully employed to target specific aldehyde oxidases of the cytochrome P450 family in cancer cells [176–179]. An array of new generation isoform-specific ALDH inhibitors are under development. Among them, duocarmycin analogues stand out due to their ultra-high alkylating potency and additional ability to specifically target ALDH1A1 [12]. Therefore, it is expected that novel, potent, and isoform-specific ALDH inhibitors could enter the pipeline of experimental and clinical assessment in cancer therapy in the coming years. Taken together, these results underscore the potential of ALDH as a therapeutic target against metastasis.

## Conclusions and future perspectives

Tumors are heterogeneous at the genetic, epigenetic, and tumorigenic level. There is substantial evidence indicating that tumor cells with stem-like capabilities are responsible, at least in part, for heterogeneity at the tumorigenic and metastatic level. ALDH stands out among the expansive and diverse group of CSC markers because of its widespread association with different types of solid tumors and the multiplicity of its biological functions, including retinoic acid signaling, antioxidant protection, osmoregulation, drug metabolism, and structural support. However, validation of ALDH as a prognostic biomarker and/or therapeutic target in the clinical setting has not yet come fully to fruition. Moving forward, it is critical that future studies include better standardization of ALDH identification and scoring methods, patient characteristics, and cohort sizes. In addition, more attention must be drawn to the study of the therapeutic effects of ALDH isoenzyme inhibitors in CTCs, DTCs, and metastatic and migratory activity. We believe that only a consistent preclinical and clinical approach revolving around CSC-mediated metastasis and therapy resistance will reveal the therapeutic and biomarker potential of ALDH in solid tumors.

In conclusion, it is clear that ALDH is not only a marker for aggressive stem-like and metastatic cells, but that it is also mechanistically involved in these behaviors. Hence, the study of ALDH as a biomarker and functional mediator of metastasis in vivo is a promissory field for discovering targets that might interfere with solid tumor progression. However, with the heterogeneity of ALDH isoforms described as CSC markers in different tumor types, and the newly described cell context dependent tumorigenic function of ALDH, it is likely that different isoforms may contribute differently to metastasis in different types of solid tumors. Moreover, given that it has been shown that other isoenzymes besides ALDH1A1 and ALDH1A3 are responsible for the activity reported in the ALDEFLUOR™ assay, and the experimental evidence supporting multi-enzyme isoform participation in the same tumor, it is also likely that more than one ALDH isoform may be contributing to progression within the same tumor. Continued intensive investigation into the functional contribution of ALDH to cancer progression and metastasis will be important for tackling the enormous therapeutic challenge that such diverse landscape imposes.
